# New Advances in the Synthetic Application of Enantiomeric 1-Phenylethylamine (α-PEA): Privileged Chiral Inducer and Auxiliary

**DOI:** 10.3390/molecules25214907

**Published:** 2020-10-23

**Authors:** Marzena Wosińska-Hrydczuk, Jacek Skarżewski

**Affiliations:** Chair of Organic and Medicinal Chemistry, Faculty of Chemistry, Wrocław University of Science and Technology, Wyb. Wyspiańskiego 27, 50-370 Wrocław, Poland; marzena.wosinska@pwr.edu.pl

**Keywords:** 1-phenylethylamines, resolution, chiral auxiliary, diastereoselectivity, chiral ligands, modular organocatalysts, asymmetric synthesis, α-methylbenzylamine

## Abstract

New developments in the synthesis, resolution, and synthetic applications of chiral 1-phenylethylamine (α-PEA) reported in the last decade have been reviewed. In particular, improvements in the synthesis of α-PEA and its derivatives and chiral resolution, as well as their applications in the resolution of other compounds, were discussed. α-PEA was used as a chiral auxiliary in the diastereoselective synthesis of medicinal substances and natural products. Chiral ligands with α-PEA moieties were applied in asymmetric reactions, and effective modular chiral organocatalysts were constructed with α-PEA fragments and used in important synthetic reactions.

## 1. Introduction

Chiral inducers and auxiliaries frequently play a key role in the practical synthesis of enantio-pure products, often drugs or agrochemicals. Introduced by A. W. Ingersoll in 1937 [1], nowadays chiral 1-phenylethylamine (α-PEA) belongs to the ancillary compounds most often used for the synthesis of enantiomeric products. This cheap primary amine, available in both enantiomeric forms, offers a structural motif frequently applied in the synthesis of chiral building blocks, useful in divergent asymmetric synthesis. Due to successful applications in various chiral recognition processes, α-PEA can be considered a privileged chiral inducer and auxiliary. Its essential synthetic applications have been included in several volumes of the encyclopedic Fieser and Fieser’s series on reagents for organic synthesis [2]. The topic was specifically reviewed by E. Juaristi in 1989 [3], 1999 [4], and 2010 [5], and briefly in 2012 by C. Kouklovsky and T. J. Wenzel [6]. However, the last decade has witnessed many interesting advancements, and over eighty new original papers have supplemented the previous works.

## 2. Improvements in the Synthesis of α-PEA and Its Derivatives

**Scheme 1:**
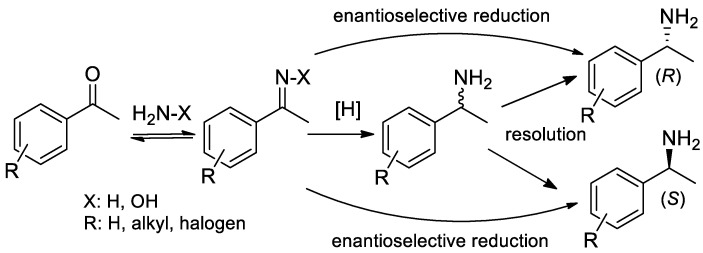
General synthetic scheme for the preparation of chiral α-phenylethylamines.

The most straightforward synthetic procedure for the preparation of α-phenylethylamine uses the reductive amination of acetophenones, and many variants of this reaction have been developed (Scheme 1) [1,2]. 

An interesting improvement to the electrochemical synthesis of α-PEA is cathode activation by introducing EDTA and pyrophosphato zinc complexes into the catholyte during the reduction of acetophenone oxime [7]. 

**Scheme 2:**
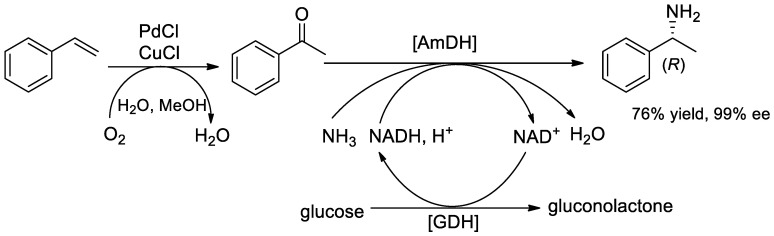
General scheme of the asymmetric hydroamination of styrene through a combination of Wacker oxidation and amine dehydrogenase (AmDH)-catalyzed reductive amination.

Recently, a new enantioselective chemo-enzymatic method for the transformation of styrene to (*R*)-α-PEA has been developed. It is based on the one-pot Pd/Cu-catalyzed Wacker oxidation of styrene to acetophenone combined with its reductive amination. Ammonia was applied as a nitrogen source, while d-glucose with glucose dehydrogenase (GDH) was used as a reductant. The catalysts were separated through membrane filtration, giving almost quantitative styrene conversion and 99% enantiomeric excess of the amine. The entire process ultimately represents the asymmetric hydroamination of styrene with ammonia (Scheme 2) [8].

## 3. Chiral Resolution of α-PEA and Its Derivatives

Essentially, both enantiomers of α-PEA are easily attained by an effective resolution of the racemate as diastereomeric salts with chiral acids, e.g., tartaric acid, *O*-functionalized natural lactic acid, and others. Further, some direct stereoselective syntheses have been reported. Despite many earlier successful resolutions, interesting observations concerning the enantioseparation of *rac*-α-PEA through diastereomeric salt precipitation were noted by the Hirose group [9]. They demonstrated that the selectivity of the diastereomeric salt formation between *rac*-α-PEA and *N*-(*p*-toluenesulfonyl)-(*S*)-phenylalanine dramatically changed from (*S*,*S*) to (*R*,*S*) with the use of solvent compounds with a six-membered ring structure, namely tetrahydropyran (THP) or cyclohexene as compared to 2-propanol. The X-ray analysis confirmed that THP was included in the (*R*,*S*) salt and stabilized it without any hydrogen bonds [9].

A simple batch procedure was developed for the solvent-free kinetic resolution of racemic primary amines by their acylation with equimolar amounts of isopropyl methoxyacetate. The process was catalyzed by *Candida Antarctica* lipase B (Novozym 435 and sol-gel CALB). *R*ac**-α-PEA was resolved giving (*S*)-α-PEA and (*R*)-α-PEA-amide with good yields and *ee* ≥ 95%. The other nine structurally different amines were resolved by this procedure with high yield and enantioselectivity [10]. 

**Scheme 3:**
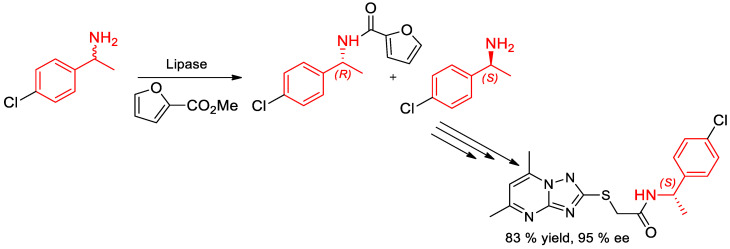
The Novozym 435-catalyzed resolution of (*R*,*S*)-1-(4-chlorophenyl)ethylamine to synthesize enantiomeric triazolopyrimidine herbicide.

Recently, for the synthesis of ring-substituted, optically active isomers of 1-phenylethylamines, the chemo-enzymatic method has also been developed [11]. The method is particularly attractive because of the commercial availability of the immobilized enzymes. Thus, for (*S*)-1-(4-chlorophenyl)ethylamine, a key intermediate for the synthesis of the corresponding acetamide (a herbicide), the resolution catalyzed by the immobilized lipase (Novozym 435) was chosen (Scheme 3) [11].

**Scheme 4:**
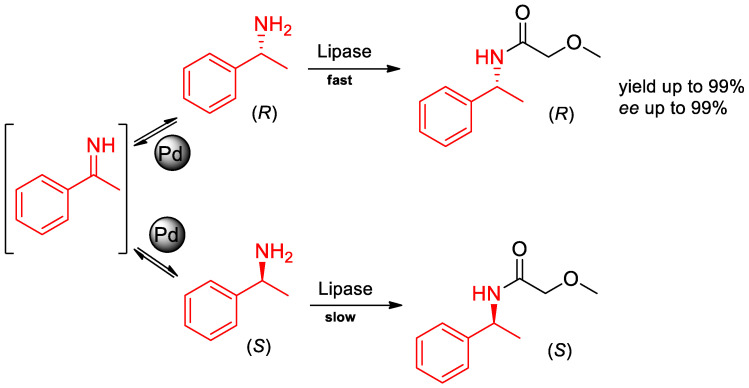
Selective dynamic kinetic resolution (DRK) of primary benzyl amines with palladium nanoparticles as the racemization (dehydrogenation–hydrogenation) catalyst and lipase as the methoxyacetylation catalysts.

Other separation procedures for α-PEA derivatives have been developed using enzymatic or chemical combined with enzymatic methods. Thus, primary benzyl amines effectively underwent a selective dynamic kinetic resolution (DRK) using palladium nanoparticles as a racemization catalyst, and lipase-catalyzed acylation with methoxyacetate ester (Scheme 4) [12].

**Scheme 5:**
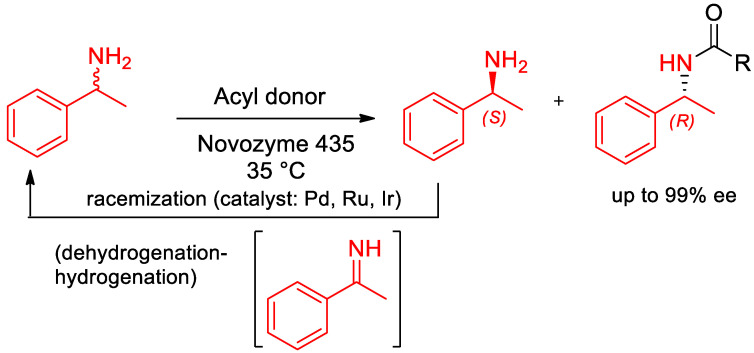
Kinetic resolution of *rac*-α-phenylethylamine under continuous flow conditions.

Furthermore, new synthetic techniques were applied in the deracemization. The continuous flow kinetic resolution of racemic α-PEA with CALB immobilized on acrylic resin (Novozyme 435) using ethyl acetate as the acylating reagent with a short residence time (40 min) gave high enantiomeric excesses. The non-acylated product was racemized (catalyzed dehydrogenation–hydrogenation) and again used for the lipase-catalyzed acylation. The process is suitable for automatization and can be scaled up for industrial purposes (Scheme 5) [13]. 

**Scheme 6:**
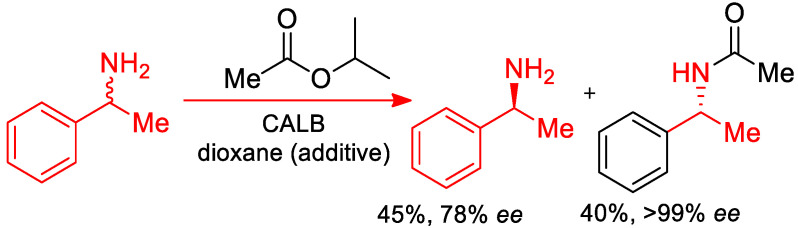
Mechanoenzymatic resolution of *rac*-α-PEA using isopropyl acetate.

For the kinetic resolution of racemic amines, another new operational synthetic technique, namely high-speed ball milling (HSBM), was used by Juaristi and coworkers [14]. The catalyzed by CALB reaction afforded acylated products with high conversion and often excellent enantiopurity (*ee* > 99%). A short reaction time was required (typically 90 min), either with 1,4-dioxane as an additive present in a minute amount or using only the acylating agent. Several acylating agents and additives were tested in the procedure, and the best outcomes resulted in isopropyl acetate as an acylating agent (Scheme 6) [14]. 

**Scheme 7:**
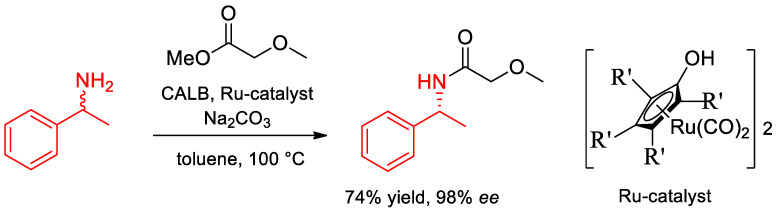
Kinetic resolution of *rac*-α-PEA.

Further, CALB-catalyzed acylation and catalyzed by ruthenium complex racemization (dehydrogenation–hydrogenation) was applied in the dynamic kinetic resolution (DKR) of *rac*-α-PEA (Scheme 7). Here, as before (Scheme 4 and Scheme 5), the Ru catalyst causes dehydrogenation–hydrogenation, thus racemizing the slowly acylated enantiomer and dynamically supplying the quickly reacting one. The reaction conditions were optimized, and alkyl methoxyacetates as acyl donors at low catalyst loadings were more effective (higher *ee*s) than isopropyl acetate [15].

**Scheme 8:**
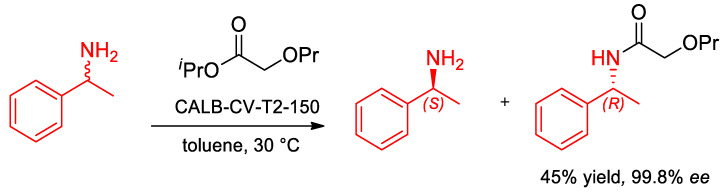
Kinetic resolution of α-PEA using isopropyl 2-propoxyacetate as an acylating agent.

The Poppe group carried out further optimization of the resolution of racemic primary amines under batch and continuous-flow conditions [16]. Particularly, they tested yield and enantiomeric excess of the *N*-acylation products obtained in the reactions catalyzed by immobilized lipase B (CALB-CV-T2-150) with isopropyl esters of several 2-alkoxyacetic acids. For *rac*-α-PEA and three other primary amines, the best results were attained using isopropyl 2-propoxyacetate. Under the optimized continuous-flow conditions, the amines underwent kinetic resolutions with high conversions and nearly quantitative enantiomeric excesses. Moreover, for five additional amines (substituted derivatives of α-PEA), 2-propoxyacetate was the preferred acylating agent, leading to the corresponding (*R*)-propoxyacetamides with very high *ee* (Scheme 8) [16].

In conclusion, the recently reported enzymatic methods for the kinetic resolution of racemic α-PEA revealed 2-alkoxyacetate esters as particularly useful acylating reagents. The lipase-catalyzed N-acylation gave the corresponding products with high conversion and excellent enantiopurity (*ee* > 99%). 

## 4. Application of Enantiomeric α-PEA in Chiral Resolution

Since both antipodes of α-PEA are so easily available and inexpensive, these compounds were often used to resolve various racemic derivatives. In particular, enantioresolution by crystallization of diastereomeric salt with enantiomeric α-PEA still enjoys much interest. This operationally simple and high-yielding methodology has been exploited and optimized for the preparation of numerous optically active acids.

Easy crystallization of the less soluble diastereomeric salt formed between (*R*)-*N*-benzyl-α-PEA and racemic 2-chloromandelic acid was used for its resolution. The structure of the grown crystals was examined in detail by X-ray analyses. Preferential host-guest interactions, stabilizing the salt of lower solubility, were interpreted by a “lock-and-key” packing and CH/π interactions within the hydrophobic moieties. The authors recommended improving the amine chiral discrimination properties by introducing a larger aromatic system to the resolving agent [17].

**Scheme 9:**
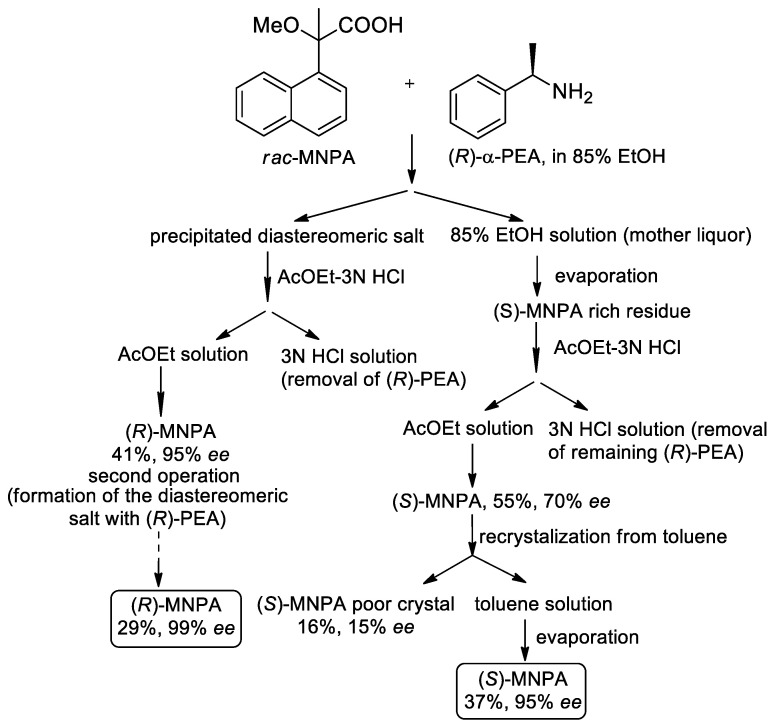
Experimental procedure for the separation of enantiomers of MNPA.

Racemic 2-methoxy-2-(1-naphthyl)propionic acid (MNPA) underwent efficient enantio-resolution with (*R*)-α-PEA, giving enantiopure (*R*)-MNPA. Thus, the precipitated crystalline salt of (*R*)-α-PEA and (*R*)-enriched-MNPA was separated, and then the acid was liberated (41%, 95% *ee*). This scalemic product was dissolved in aqueous ethanol and treated again with (*R*)-α-PEA to give the corresponding salt. This salt, after acidification, afforded 29% of (*R*)-MNPA (>99% *ee*). A mother liquor from the first crystallization was evaporated, and (*S*)-MNPA (95% *ee*) was finally isolated by a sequential crystallization. The X-ray analysis uncovered four independent hydrogen bonds and one CH/π interaction stabilizing the first precipitated diastereomeric salt (Scheme 9) [18]. 

**Scheme 10:**
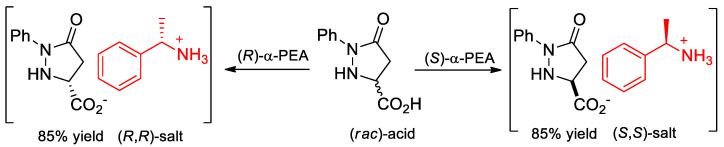
(*R*)- and (*S*)-α-PEA resolution of 5-oxo-1-phenylpyrazolidine-3-carboxylic acid.

Furthermore, racemic 5-oxo-1-phenylpyrazolidine-3-carboxylic acid was resolved into enantiomers. The racemate was treated with a half equivalent of (*R*)-α-PEA, giving mostly (*R,R*)-salt precipitated from the solution. The remaining part, enriched in the *(S*)-acid, was worked up with (*S*)-α-PEA, as shown in Scheme 10 [19]. 

**Scheme 11:**
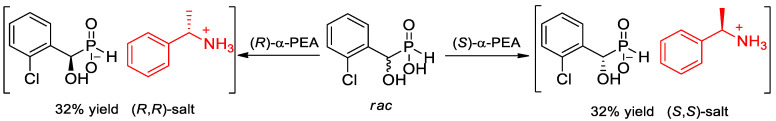
(*R*)- and (*S*)-α-PEA resolution of *rac*-α-hydroxy-(o-chlorophenyl)methyl]phosphinic acid.

Investigation of the resolution of racemic α-hydroxy-(*o*-chlorophenyl)methyl]phosphinic acid with (*R*)-α-PEA via diastereomeric salt formation revealed that the (*R,R*)-salt precipitation from 2-propanol was an efficient resolving method for obtaining a single enantiomer of phosphinic acid. Resolving *rac*-phosphinic acid with (*S*)-α-PEA in EtOH by the same procedure gave access to (*S*)-acid in 32% yield (Scheme 11) [20]. 

**Scheme 12:**
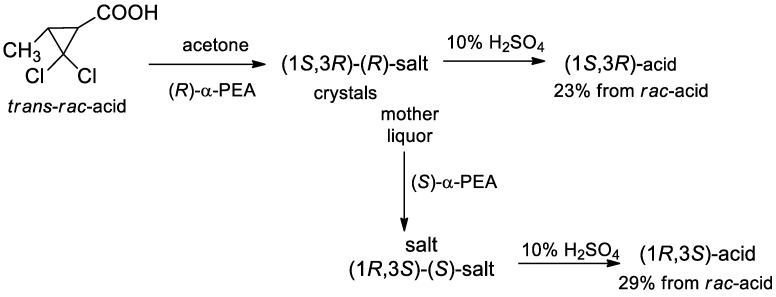
Optical resolution of *rac-trans*-2,2-dichloro-3-methylcyclopropanecarboxylic acid.

The optical resolution of *rac-trans*-2,2-dichloro-3-methylcyclopropanecarboxylic acid was reported by Kovalenko and Kulinkovich [21]. Thus, the racemic acid in acetone treated with (*R*)-α-PEA precipitated the diastereomericaly enriched (1*S*,3*R*)-(*R*)-salt, which after two recrystallizations from 90% aqueous acetone and subsequent treatment with dilute sulfuric acid gave the levorotary enantiomer (1*S*,3*R*)-acid in 23% yield. The remaining mother liquor and (*S*)-α-PEA gave the respective salt, that after acidification gave (1*R*,3*S*)-acid in 29% yield (Scheme 12) [21].

**Scheme 13:**
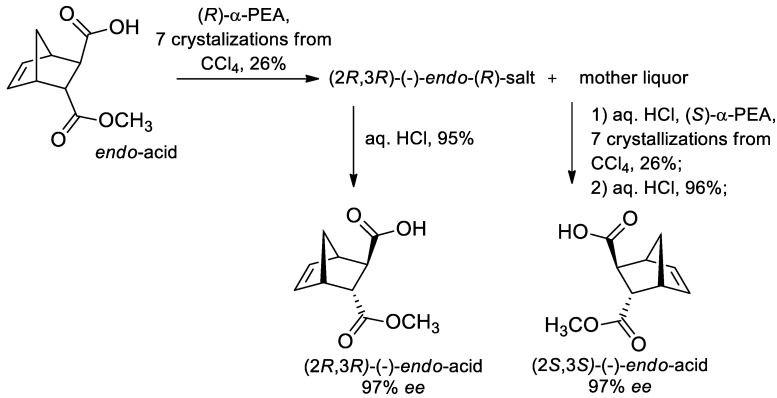
Separation of enantiomers of *trans*-norborn-5-ene-2,3-dicarboxylic acid mono-methyl ester into enantiomers.

Similarly, enantiomers of *trans*-norborn-5-ene-2,3-dicarboxylic acid mono-methyl ester were separated by crystallization of the respective salts with (*R*)- and (*S*)-α-PEA (Scheme 13) [22]. Both simple procedures produced interesting chiral building blocks for further synthesis.

**Scheme 14:**
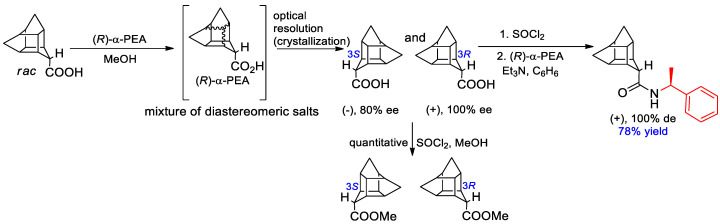
Optical resolution of *D*_3_-symmetric tris-homocubane-4-carboxylic acid.

Volochnyuk and coworkers reported a multigram synthesis of *D*_3_-symmetric tris-homocubane-4-carboxylic acid. Its optical resolution was achieved through the recrystallization of salt with (*R*)-α-PEA. The absolute configuration (*R*) of pure (+)-enantiomeric form was assigned by X-ray crystal structure analysis (Scheme 14) [23].

**Scheme 15:**
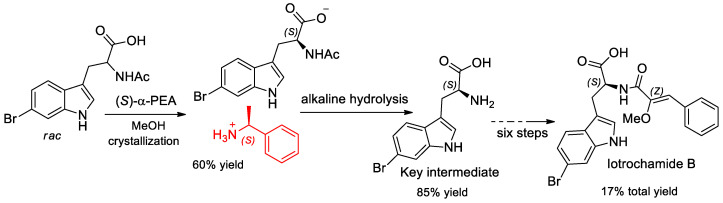
Optical resolution of *rac*-6-bromo-N-acetyltryptophan, intermediate in the synthesis of Iotrochamide B.

In 2019, Zhao and coworkers published the synthesis of natural Iotrochamide B, earlier isolated from the marine sponge *Iotrochota* sp. A key intermediate in the synthesis was (*S*)-6-bromo-tryptophan obtained from the corresponding racemic *N*-acetyltryptophan by its resolution with (*S*)-α-PEA (Scheme 15) [24].

**Scheme 16:**
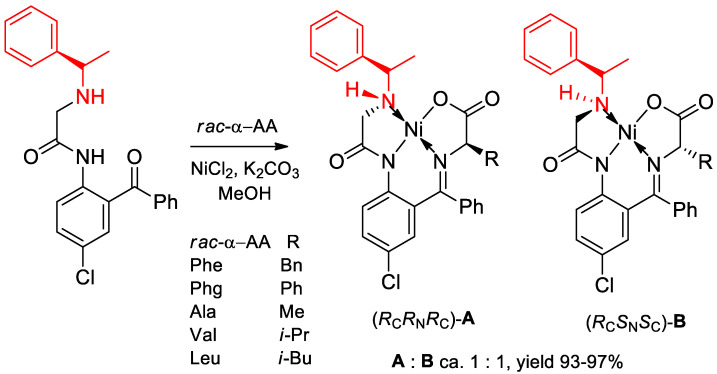
Reactions of the ligand derived from (R)-α-PEA with racemic α-amino acids.

An interesting resolution of α-amino acids has been developed using reagents derived from α-PEA. Thus, the group of Soloshonok reported that chirally modified ketone reacts with racemic α-amino acids (*rac*-α-AAs) in nearly quantitative yield, giving two major and two minor diastereomeric nickel complexes. Thus, the complexes (*R*_C_,*R*_N_,*R*_C_)-**A** (major) along with (*R*_C_,*S*_N_,*R*_C_, minor) coming from (*R*) α-amino acids, and (*R*_C_,*S*_N_,*S*_C_)-**B** (major) along with (*R*_C_,*R*_N_,*S*_C_, minor) derived from α-amino acids of (*S*) configuration were formed in nearly equal amounts and excellent yield. The major complexes **A** and **B** were easily separated and gave the corresponding pure enantiomers of the amino acids. The resolving agent’s recovery and recycling make the complete separation process very attractive (Scheme 16) [25].

**Scheme 17:**
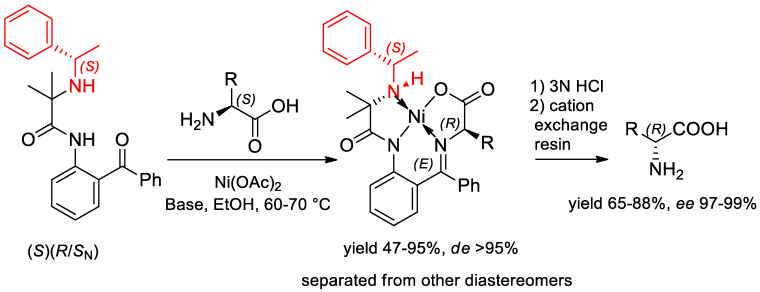
Conversion of (*S*)-amino acids to (*R*)-amino acids.

The same group designed and synthesized a similar ligand to convert natural (*S*)-α-amino acids into the unnatural (*R*)-enantiomers. They prepared (*S*)-α-PEA-derived benzophenone forming the respective Schiff bases with various (*S*)-α-amino acids. After Ni(II) complexation, epimerization at the amino acid stereogenic center occurred. The formation of the new diastereomeric complex took place in a thermodynamically controlled manner. Later, the pure diastereomeric complex was hydrolyzed, and the chiral benzophenone and resulting (*R*)-amino acid were isolated by cation exchange chromatography (Scheme 17) [26].

**Scheme 18:**
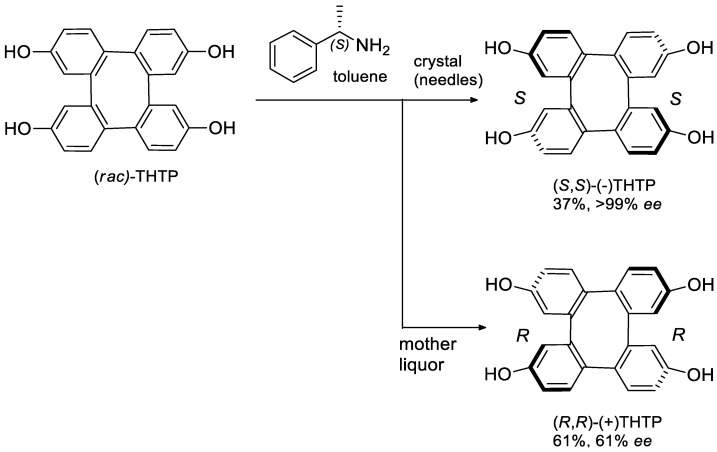
Enantiomeric enrichment of (*S*,*S*)-(−)-THTP using (*S*)- α-PEA.

An interesting option for the resolution of a racemic mixture of host molecules is the formation of the respective inclusion complexes with an enantiomeric guest, here α-PEA. This idea was applied by the group of Kaku and Tsunoda to carry out the optical resolution of *rac*-tetrahydroxytetraphenylene (THTP) to afford (*S*,*S*)-THTP in good yield and high *ee* (Scheme 18). They also obtained (*R*,*R*)-THTP using (*R*)-α-PEA. The thermal analyses (TGA and DSC) suggested that the *S,S*-*S* complex was a less stable clathrate than the *S,S*-*R* complex [27].

**Scheme 19:**
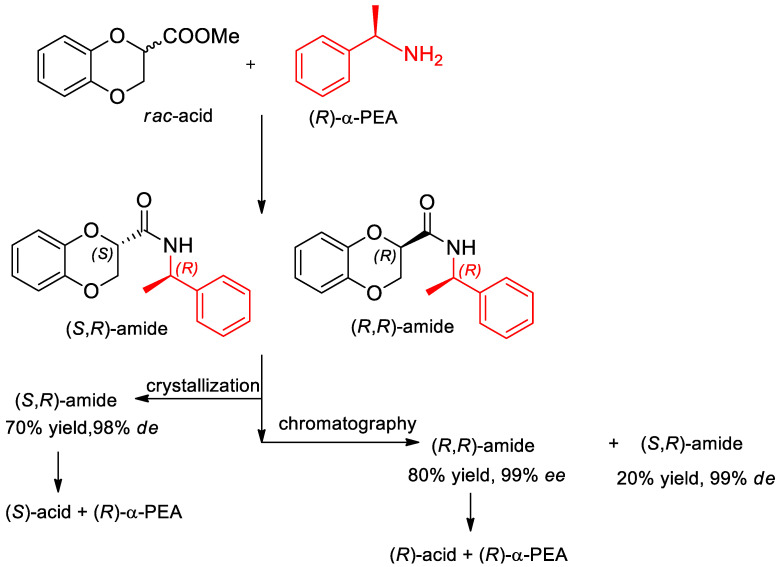
Resolution of racemic 1,4-benzodioxane-2-carboxylic acid via diastereomeric phenylethylamides.

When diastereomeric α-phenylethylammonium salts do not differ enough, the simple resolution of racemic acid is impossible. In that case, the corresponding diastereomeric amides can be successfully used. This strategy has been applied for the highly effective resolution of racemic 1,4-benzodioxane-2-carboxylic acid by precipitation of the less soluble amide diastereomer of *unlike* configuration (>98% de) (Scheme 19) [28]. 

**Scheme 20:**
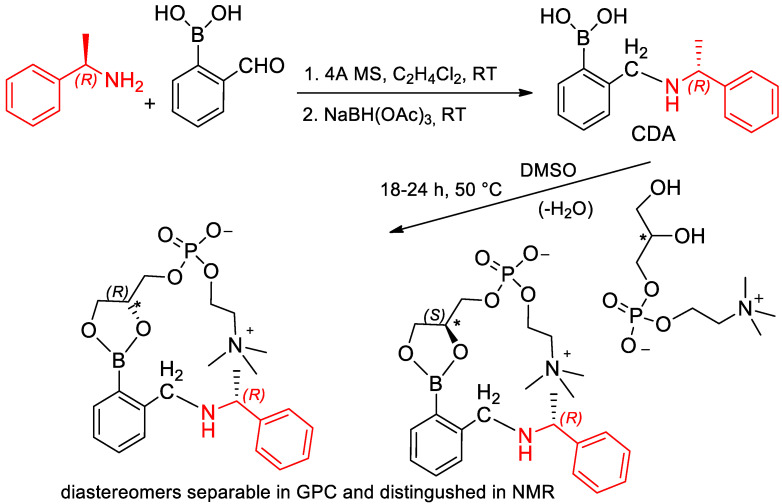
Synthesis and analytical application as a chiral derivatizing agent (CDA) of α-PEA-containing chiral boronic acid.

The determination of the enantiomeric purity of various chiral compounds can be achieved by applying chiral derivatizing agents (CDAs). The Bull group developed α-PEA-containing chiral boronic acid as a CDA to determine the enantiomeric purity of vic-diols [29]. Recently, de Ferra and coworkers [30] described a similar procedure for the determination of enantiomeric excess of α-glycerophosphocholine with a CDA prepared according to Anslyn’s procedure (Scheme 20) [31]. 

**Scheme 21:**
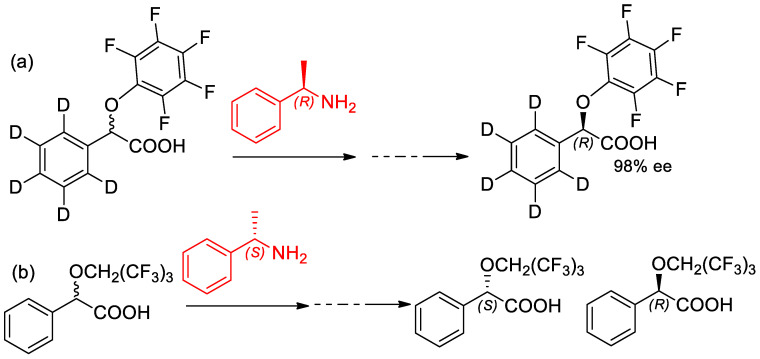
Resolution of fluoro-derivatized mandelic acid as a chiral solvating agent (CSA): (**a**) the resolution of (*rac*)-2-(pentafluorophenoxy)-2-(phenyl-*d*_5_)acetic acid, (**b**) the resolution of (*rac*)-α-(nonafluoro-tert-butoxy)carboxylic acid.

Highly demanded chiral fluoro-compounds find application as chiral solvating agents (CSAs) in ^1^H and ^19^F NMR. Cavalluzzi and collaborators obtained (−)-(*R*)-2-(pentafluorophenoxy)-2-(phenyl-*d*_5_)acetic acid (Scheme 21a) of (98% *ee*) by resolution of a racemate with (−)-(*R*)-α-PEA. The obtained chiral CSA was successfully used for the direct ^1^H NMR determination of enantiomeric excess of chiral quinoline-containing antimalarial drugs, namely mefloquine, chloroquine, and hydroxychloroquine [32]. 

Similarly, Béni’s group resolved α-(nonafluoro-*tert*-butoxy)carboxylic acid using enantiomeric (*S*)-α-PEA (Scheme 21b) [33]. The enantioenriched fluoro-derivatized CSA gave ^19^F NMR chemical shift differences (Δδ) 0.006–0.028 ppm in CDCl_3_ and 0.010 and 0.014 ppm in apolar C_6_D_6_ with biologically active amines [33].

**Scheme 22:**
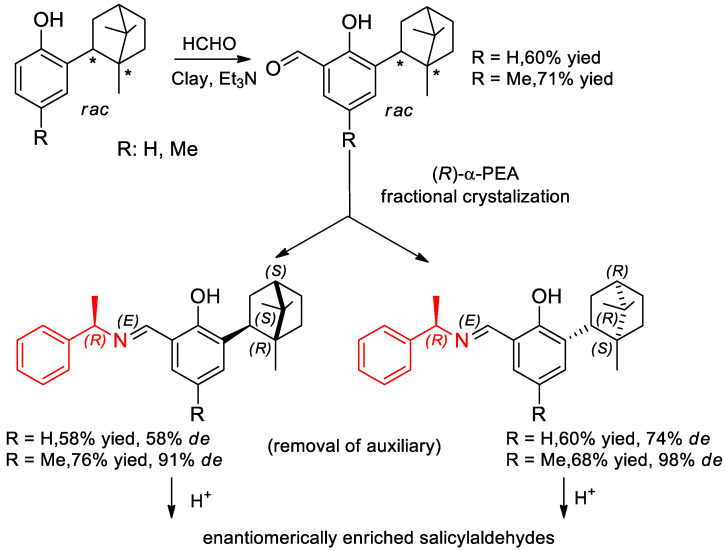
Resolution of racemic salicylaldehydes containing an isobornyl substituent.

A different synthetic approach was used for the resolution of racemic salicylaldehydes containing an isobornyl substituent. The aldehydes were converted with (*R*)-α-PEA into the corresponding Schiff bases, and the diastereomeric imines were separated by fractional crystallization from pentane. Diastereomers were hydrolyzed, giving enantioenriched aldehydes along with the recovered chiral auxiliary (Scheme 22) [34]. 

**Scheme 23:**
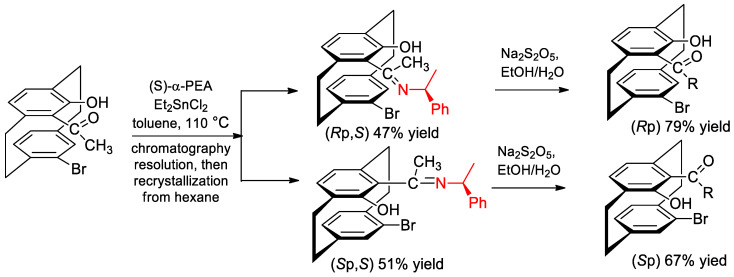
Resolution of 4-acyl-13-bromo-5-hydroxy[2.2]paracyclophane into enantiomers.

Vorontsova and coworkers elaborated a procedure for the synthesis of ligands based on 4-acyl-13-bromo-5-hydroxy[2.2]paracyclophane. The enantiomers of planar chiral ketones were reacted with (*S*)-α-PEA in the catalytic presence of Et_2_SnCl_2,_ giving nearly quantitative amounts of an equimolar mixture of two diastereomeric Schiff bases. Their separation and hydrolysis furnished an enantiomer (for the acetyl-derivative) (*R*p) (${\left[\alpha \right]}_{D}^{20}$ + 363.3, toluene) in 79% yield and (*S*p), (${\left[\alpha \right]}_{D}^{20}$ − 361.5, toluene), with 67% yield (Scheme 23) [35].

## 5. Enantiomeric Enrichment of α-PEA and Its Derivatives—Self-Disproportionation of Enantiomers (SDE) 

When partially non-racemic samples are crystallized or undergo chromatography, often enantiomeric molecules act vs. the aggregate of both enantiomers (racemate) as diastereomers. In effect, under totally achiral external conditions, the scalemic samples are additionally enantioenriched. This process is known as the self-disproportionation of enantiomers (SDE) [36]. The SDE effect has also been observed for the amides of α-PEA. Thus, the medium-pressure liquid chromatography of non-racemic acetamide derivatives showed clear splitting into two separate fractions, both corresponding to the acetamide. For the material of the less polar fraction, significant enantiomeric enrichment was observed. Simultaneously, the more polar fraction material had the *ee* value considerably reduced [37,38,39]. The SDE effects were also described in achiral column chromatography of a series of *N*-fluoroacetylated α-PEA-amides [40]. The SDE consequences, potential problems, and prospective applications have recently been reviewed and discussed [41].

## 6. α-PEA as a Chiral Auxiliary in Diastereoselective Synthesis

### 6.1. Synthesis of Compounds with Biological Activity

The synthesis of compounds with the prospective biological activity in the pure enantiomeric form is of particular interest in medicinal chemistry. Quite often, only one specific stereoisomer is responsible for the therapeutic effect, whereas the other can cause harmful side effects. In this context, a wide variety of compounds have been synthesized using α-PEA as a chiral auxiliary or a building block. 

**Scheme 24:**
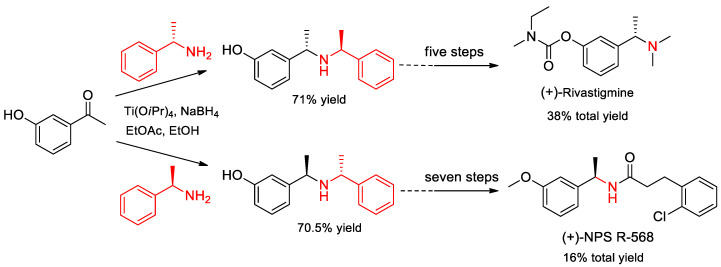
Syntheses of Rivastigmine and NPS R-568.

In line with this general goal, an economically viable strategy for the synthesis of (+)-Rivastigmine and (+)-calcimimetic NPS R-568 has been devised by Rao and collaborators (Scheme 24) [42]. The method was based on the reductive amination of 3′-hydroxyacetophenone with (*S*)-α-PEA or (*R*)-α-PEA in the presence of titanium(IV)isopropoxide and NaBH_4_. The α-PEA moieties were cleaved in regioselective hydrogenation of the bis amines to yield the required chiral amines with excellent enantioselectivity. 

**Scheme 25:**
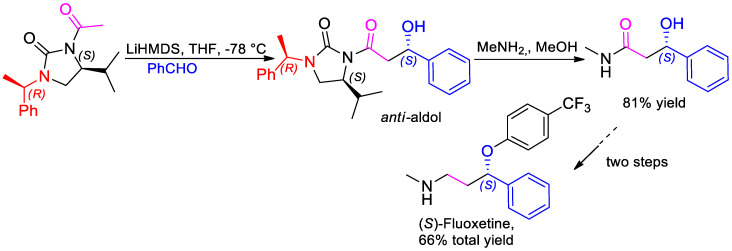
Stereoselective synthesis of (*S*)-Fluoxetine.

Nair and coworkers explored the acetate aldol reaction designed for the stereoselective synthesis of (*S*) and (*R*)-Fluoxetine [43]. This compound in racemic form is a potent, selective serotonin reuptake inhibitor, whereas the separated enantiomeric forms have different therapeutic effects. The synthesis started from the acetate aldol reaction of *N*-acetyl-(*S*)-4-isopropyl-1-[(*R*)-1-phenylethyl]imidazolidin-2-one. The product was converted into (*S*)-3-hydroxy-*N*-methyl-3-phenyl propanamide. After reduction and aromatic substitution on 4-fluorobenzotrifluoride, (*S*)-fluoxetine was obtained in enantiopure form and excellent yield. (*R*)-Fluoxetine was obtained in a similar procedure (Scheme 25). With (*R*) or (*S*)-α-PEA moiety, the aldol reaction gave a high yield and anti-aldol selectivity. Interestingly, the configuration of α-PEA did not influence the product’s stereochemistry as both enantiomers of this group afforded similar yields and the same selectivity. Moreover, the placement of benzylamine or *tert*-butylamine moieties instead of α-PEA resulted in yield and selectivity deterioration. The authors ascribed the influence of α-PEA to the stereoelectronic effects, enhancing the enolate reactivity [43].

**Scheme 26:**
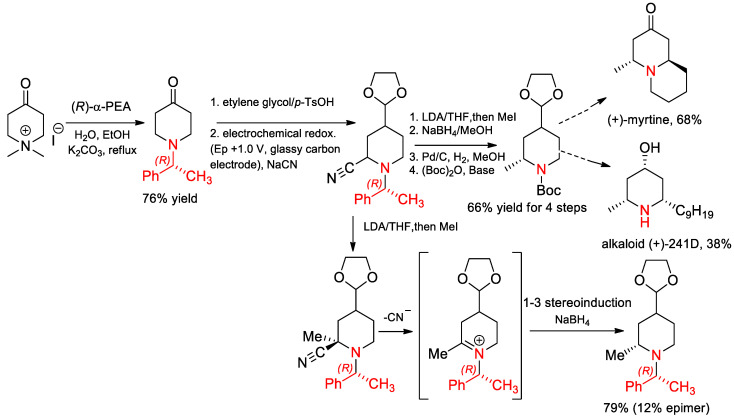
General scheme for the synthesis of (+)-myrtine and (+) alkaloid 241D.

The total synthesis of *trans*-quinolizidine derivatives, namely (+)-myrtine and *cis*-2,4,6-trisubstituted piperidone alkaloid (+)-241D, was described by Hurvois and coworkers [44]. The first step was performed according to the procedure reported earlier by Pawłowska and Czarnocki [45] (see below), and here α-PEA allowed an efficient 1–3 stereoinduction. Then, the key intermediate was transformed by different steps, giving (+)-myrtine and alkaloid(+)-241D with a total yield of 68 and 38%, respectively (Scheme 26). 

**Scheme 27:**
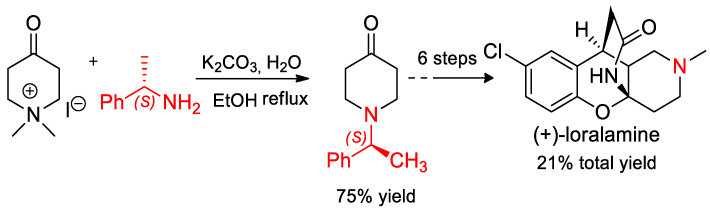
(S)-α-PEA as a chiral auxiliary in the synthesis of (+)-lortalamine.

The Czarnocki group developed the synthesis of (+)-lortalamine, starting with (*S*)-α-PEA as a chiral auxiliary [45]. The method was based on the preparation of *N*-[(*S*)-α-phenylethyl]-4-piperidone (Scheme 27), a procedure advanced earlier by Kuehne et al. [46]. The corresponding nucleophilic substitution (ammonium salt + amine) results in a ring opening–ring closure sequence, leading to the key chiral intermediate.

**Scheme 28:**
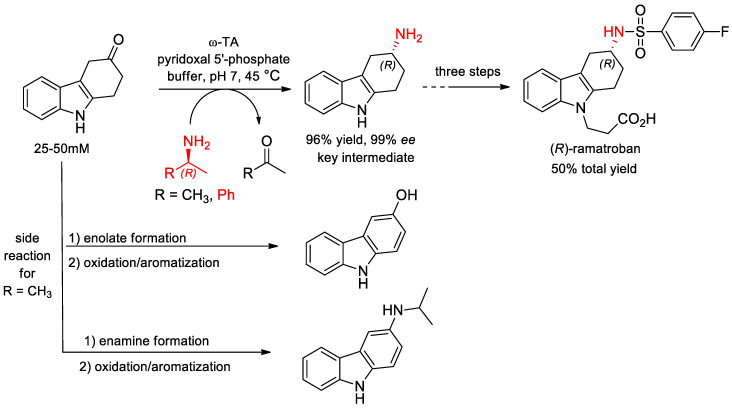
Synthesis of the enantiopure (*R*)-2,3,4,9-tetrahydro-1*H*-carbazol-3-amine, a key intermediate for the preparation of (*R*)-ramatroban.

α-PEA is also used as an important chiral auxiliary in the synthesis of (*R*)-ramatroban [47]. This sulfonamide was developed to treat allergic rhinitis and asthma and could have a therapeutic effect on coronary artery diseases. The amination was performed using ω-transaminases (ω-TA). Correspondingly, the most important synthetic step is the ω-TA-catalyzed transformation of the ketone to enantiopure (*R*)-2,3,4,9-tetrahydro-1*H*-carbazol-3-amine. In this reaction, (*R*)-α-PEA was used as an amine donor. In that case, only the desired product was obtained, whereas with 2-propylamine, various unwanted side products were formed (Scheme 28).

**Scheme 29:**
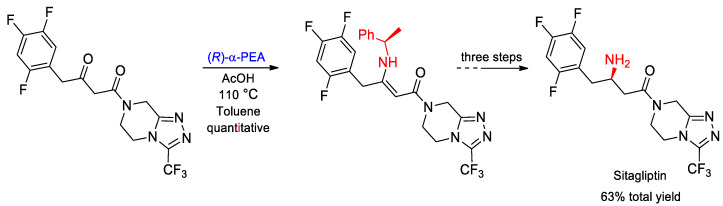
Synthetic route to Sitagliptin.

A practical asymmetric synthesis of Sitagliptin, an approved drug for treating type 2 diabetes mellitus, was elaborated by the Kozlowski and Bandichhor groups [48]. The adopted approach used α-PEA as a chiral auxiliary, allowing for stereoinduction in the reductive amination (Scheme 29). This strategy offers a very simple diastereoselective and efficient procedure.

**Scheme 30:**
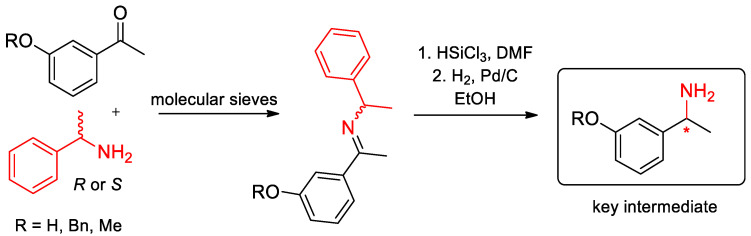
Reductive amination in the synthesis of a key intermediate for the synthesis of compounds with biological activity.

In 2017, Brenna described the procedure for the synthesis of both enantiomers of 1-(*m*-hydroxyphenyl)ethylamine, an important intermediate for the preparation of valuable active pharmaceuticals [49]. The procedure was based on using (*R*)- or (*S*)-α-PEA-imines as chiral building blocks. The trichlorosilane reduction of the imines followed by selective hydrogenolysis under batch as well as flow conditions gave the desired product with almost complete stereochemical induction and high yields (Scheme 30). This product is a key intermediate in the synthesis of compounds with pharmaceutical properties. The same group also reported applying a similar method for the preparation of the intermediate for the other biologically important compounds, such as Rasagiline and Tamsulosin [50]. In that case, the cyclopentadienone-based iron complexes were used to catalyze the diastereoselective hydrogenation of enantiopure α-PEA-imines successfully.

**Scheme 31:**
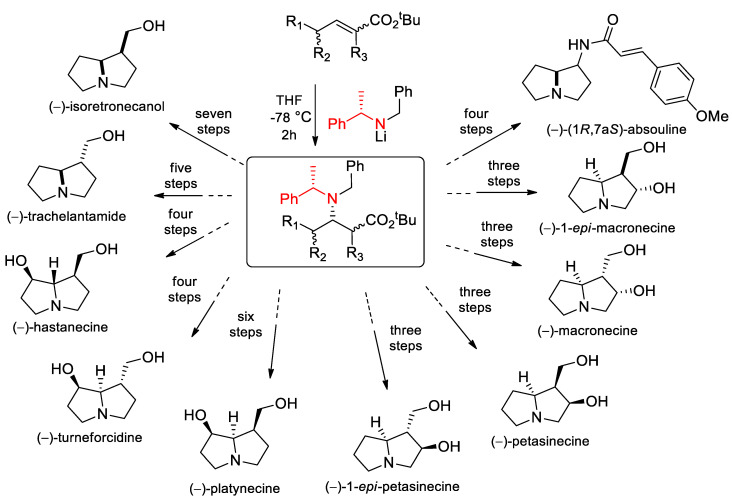
The Davis’ syntheses of substituted pyrrolizidine derivatives.

Natural pyrrolizidine alkaloids consist of two fused five-membered rings with a nitrogen atom at the bridgehead (Scheme 31). Many of them have interesting biological activities. Davies and coworkers have developed an effective method for the synthesis of a collection of pyrrolizidine alkaloids, starting with the preparation of (−)-(1*R*,7a*S*)-absouline [51]. The method is based on the diastereoselective conjugate addition of lithium (*S*)-*N*-benzyl-*N*-(α-methylbenzyl)amide to an enantiopure α,β-unsaturated ester derived from L-proline (Scheme 31). Later, the same strategy was applied for the synthesis of (−)-isoretronecanol and (−)-trachelantamidine [52] (Scheme 31). Following the elaborated synthetic methodology, this research group obtained the next compounds of this class [53,54] (Scheme 30). 

### 6.2. Synthesis of Imides 

Generally, the most often used chiral auxiliaries rely on their cyclic structure (e.g., Evans’ oxazolidinones, Oppolzer’s sultams, or SAMP/RAMP compounds) that limit the presence of different conformers that could diminish the selectivity. For example, see Scheme 25, [43]. However, acyclic chiral auxiliaries are also of interest. Their flexibility may allow for the reactions to be hampered by the stiffness of the established cyclic auxiliaries. 

**Scheme 32:**
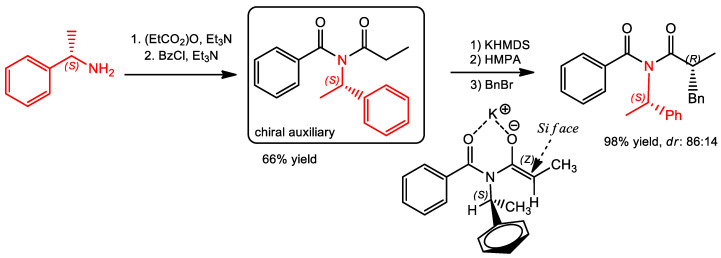
Synthesis of acyclic chiral auxiliary and its use in the alkylation reaction.

Hernández-Rodríguez, looking for a new acyclic auxiliary, described interesting diastereoselectivity in the α-alkylation of chiral propionimide [55]. The reaction of (*S*)-α-PEA as with propionyl anhydride and then benzoyl chloride gave a chiral auxiliary. The obtained compound was used in the alkylation of the prochiral fragment to form, diastereoselectively, the respective chiral imide (Scheme 32). The selectivity was due to the chelate structure with the carbonyl of the auxiliary and the allylic 1,3-strain of the amide that froze the conformation between the stereocenter of the auxiliary and the nitrogen.

### 6.3. α-PEA and Its Derivatives in the Diastereoselective Cyclization Reaction 

**Scheme 33:**
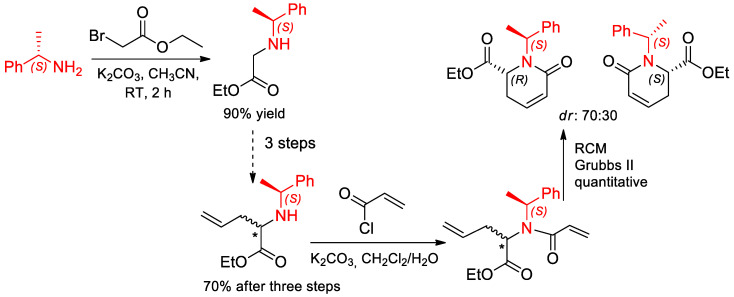
Synthesis of α,β-unsaturated piperidin-2-ones.

Synthesis of chiral heterocyclic compounds via cyclization constitutes an important part of organic chemistry. Stereoselective cyclizations are often key reactions in the synthesis of natural products. α-PEA and its derivatives have recently been used as chiral auxiliaries in the corresponding diastereoselective reactions. These processes were applied to obtain piperidin-2-ones, lactams, imidazole, and indole derivatives, the compounds expected to exhibit biological activities. Thus, in 2014, Terán and coworkers described an effective procedure for the synthesis of cyclic α-amino acids, specifically 4-substituted piperidine-2-carboxylic acid and its derivatives with pharmacological activity [56]. The procedure was based on ring-closing metathesis (RCM) to build the α,β-unsaturated piperidin-2-ones derived from (*S*)-α-PEA (Scheme 33). 

**Scheme 34:**
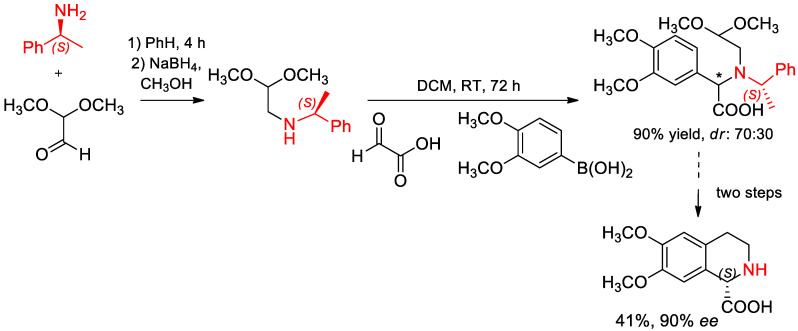
Synthesis of (+)-6,7-dimethoxy1,2,3,4-tetrahydroisoquinoline-1-carboxylic acid.

Another strategy for the synthesis of cyclic compounds is using the Pomeranz–Fritsch–Bobbitt and the Petasis reactions. The Rozwadowska group used this method for the preparation of (+)-6,7-dimethoxy1,2,3,4-tetrahydroisoquinoline-1-carboxylic acid (90% *ee*) [57]. In the first step, dimethoxyacetaldehyde reacted with chiral amines such as (*S*)-α-PEA, followed by a NaBH_4_ reduction at reflux to give the appropriate acetate in 90% yield. Then, these compounds were transformed into the appropriate acid (Scheme 34) [57]. 

**Scheme 35:**
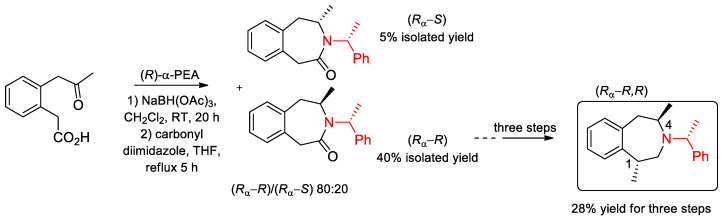
Synthesis of chiral benzoazepines which can interact with σ1 receptors.

Other important compounds synthesized by cyclization are chiral tetrahydro-3-benzazepines. These derivatives interact with σ1 receptors, which play a key role in controlling the activity of various ion-channels and neurotransmitter systems. Therefore, the synthesized compounds are prospective psychoactive drugs. In their synthesis, the important first step required enantiomerically pure (*R*)-α-PEA as a chiral auxiliary for the formation of diastereomeric lactams in 80:20 dr (Scheme 35) [58].

**Scheme 36:**
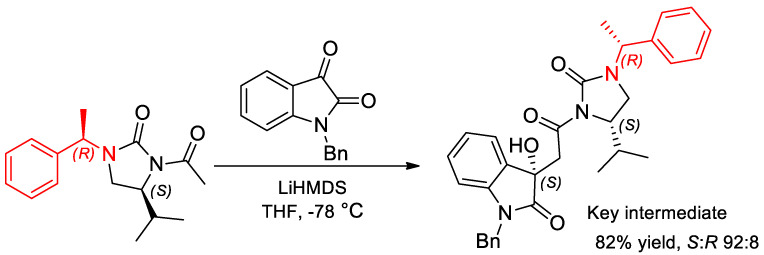
*Syn*-selective acetate aldol reaction of chiral imidazolidin-2-one.

3-Substituted-3-hydroxy-2-oxindoles are key intermediates for the synthesis of furoindolines and pyrroloindole-based natural products [59]. Their synthesis was based on the application of an α-PEA-containing chiral auxiliary. The acetate aldol reaction, which was developed earlier by the same group [43], when run with *N*-substituted isatin, afforded the corresponding adducts with high yields and diastereoselectivity (Scheme 36) [59].

**Scheme 37:**
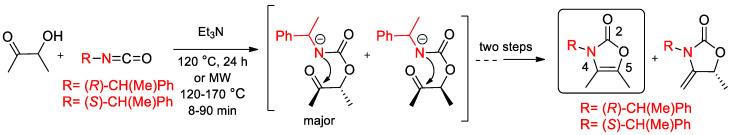
Thermal and microwave (MW)-promoted synthesis of oxazolin-2-ones.

Recently, chiral 1,3-oxazolidin-2-ones have been devised as an efficient chiral auxiliary to synthesize carboxylic compounds [60]. Consequently, (*R*)- or (*S*)-α-PEA fragment was used for the diastereoselective induction in the 4-oxazolin-2-one scaffold at the C-4 and C-5 stereocenters (Scheme 37).

### 6.4. Asymmetric Aminocarbonylation of Iodoalkenes 

**Scheme 38:**
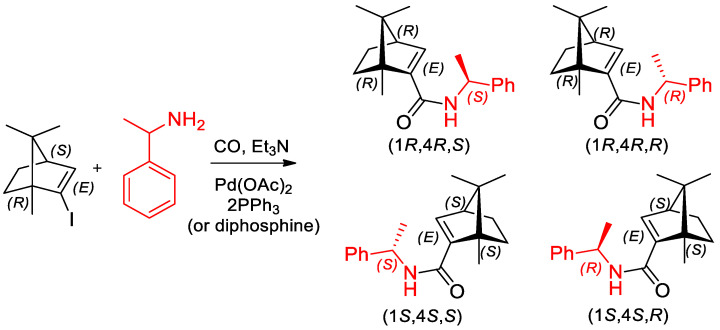
Aminocarbonylation of 2-iodobornene with α-PEA.

The palladium-catalyzed aminocarbonylation of alkenyl iodides enjoys interest as an important method for the introduction of new carbonyl functionality in organic compounds. Kollár’s group described the stereochemistry of products obtained in the asymmetric aminocarbonylation of iodoalkenes [61]. They presented the reaction of chiral iodoalkenes with α-PEA in the presence of Pd catalyst. All possible epimers were synthesized and fully characterized. However, very low (mostly negligible) diastereoselectivities were observed (Scheme 38).

### 6.5. Multicomponent Amino Alkylations 

Multicomponent amino alkylations are preferentially used to build two new bonds in one step. Primary amines reacting with carbonyl derivatives (aldehydes or ketones) form reactive Schiff bases, to which, in turn, various nucleophiles can be added. 

**Scheme 39:**
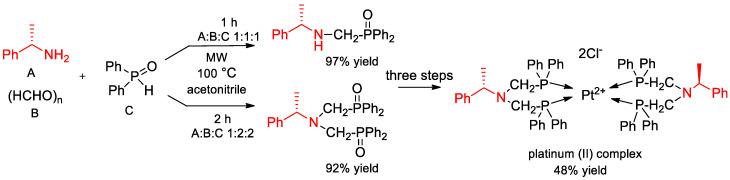
Synthesis of mono- and bis-phosphine oxides and phosphine with chiral α-PEA moiety.

The Kabachik–Fields condensation of aldimines is one of the most commonly used reactions to prepare α-aminophosphonate derivatives. Recently, a solvent-free method for the synthesis of optically active α-aminophosphonates has been described [62]. The authors used microwave (MW)-assisted Kabachnik–Fields condensations of (S)-α-PEA (A), paraformaldehyde (B), and various R_2_P(O)H (C) reagents. Depending upon the A/B/C molar ratio (1:1:1 or 1:2:2) of substrates, either (S)-diphenyl-α-phenylethylaminomethylphosphine oxide or (S)-bis(diphenylphosphinoylmethyl)- α-phenylethylamine was obtained as a product (Scheme 39). The products were isolated in 97% and 92% yields, respectively. After deoxygenation with phenylsilane, the optically active bis-phosphine was converted to chiral platinum(II) complexes, including an unusual tetradentate structure [62]. 

**Scheme 40:**
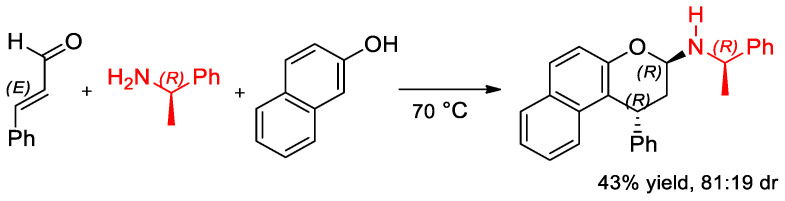
Three-component aromatic Betti-type reaction.

Since the preparation of single stereoisomers of aminoalkylation products is of considerable synthetic importance, there is still much interest in new gentle and direct approaches, including applying the Mannich reaction and Betti synthesis. Palmieri described a short synthesis of benzo[f]chromen-3-amine derivatives using a three-component aromatic Betti-type reaction [63]. The enantiomerically pure products were obtained from β-naphthol, chiral α-PEA, and α,β-unsaturated aldehydes (Scheme 40) [63].

**Scheme 41:**
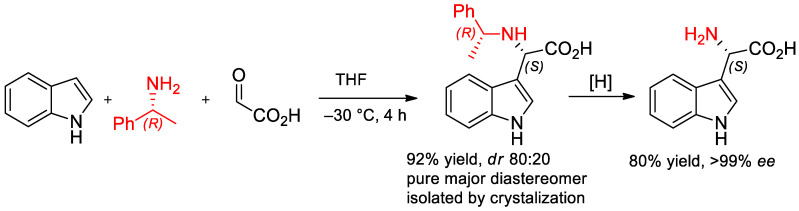
Three-component Mannich-type reaction gave the (*R,S*)-diastereomer, transformed by selective N-debenzylation to enantiopure (*S*)-indolylglycine.

Another three-component Mannich-type synthesis was developed for the simple preparation of enantiomerically pure 3-indolylglycines (Scheme 41). Despite the medium diastereoselectivity of the Mannich reaction (80/20 dr), the pure diastereomer was easily separated, and the selective *N*-debenzylation was performed with triethylammonium formate catalyzed by Pd/C, resulting in the enantiomerically pure (*S*)-indolylglycine [64].

### 6.6. Novel Derivatives of α-PEA. Synthesis of Enantiomeric Guanidines and Guanidinium Salts 

**Scheme 42:**
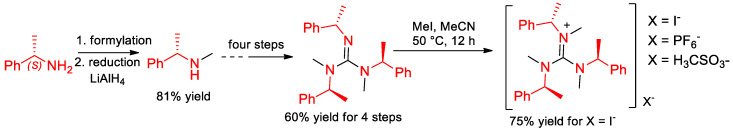
Synthesis of chiral (*S*,*S*,*S*)-tris-(1-phenylethyl)guanidine and guanidinium salts from (*S*)-α-PEA.

Guanidines and guanidinium salts are often used in organic synthesis (strong bases, catalysts, or ionic liquids) and also constitute a part of natural compounds (L-arginine, polycyclic marine alkaloids). Jäger and coworkers described the new efficient synthesis of chiral guanidines and guanidinium salts based on optically active α-PEA (Scheme 42) [65]. Unfortunately, the attempted catalytic use of the obtained guanidine and guanidinium iodide in asymmetric transformations (Morita–Baylis–Hillman, Michael, and epoxidation reactions) did not result in a noteworthy stereoinduction [65].

### 6.7. Novel Derivatives of α-PEA. Synthesis of Enantiomeric Derivative of Imidazole N-Oxides

**Scheme 43:**
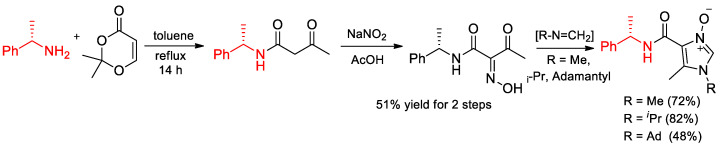
Synthesis of imidazole N-oxides.

Chiral amines, including α-PEA, are often used as a starting material for the synthesis of novel, optically active heterocyclic systems. The products are designed as the prospective chiral ligands. For this purpose, an efficient method for preparing enantiomeric imidazole *N*-oxides was developed by Mlostoń and coworkers [66]. Chiral acetylacetate amide was prepared by the reaction of acetylketene with (*S*)-α-PEA. Subsequent nitrosation of β-oxoamide led to the intermediate being transformed into enantiomerically pure 3-oxidoimidazole-4-carboxamides with good yield (Scheme 43).

### 6.8. α-PEA in the Strecker Reaction 

**Scheme 44:**
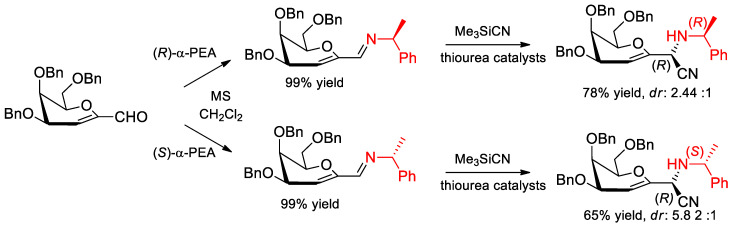
Preparation of d-galactal-linked α-aminonitriles via preformed galactal imines.

An interesting study on the stereochemistry of the synthesis of new 2-amino-2-*C*-D-glycosyl-acetonitriles was carried out using the Strecker reaction. The products are important building blocks for synthetic carbohydrate chemistry. Various *C*-glycosyl aldehydes were reacted with (*R*)- or (*S*)-α-PEA to afford the respective Schiff bases. The thiourea-catalyzed reaction with a cyanide donor occurred at re-face in both cases, and the nucleophilic addition of HCN gave predominantly *R*-configured *C*-glycosyl aminoacetonitriles. Higher diastereoselection was observed for (*S*)-α-PEA-imine than for the (*R*)-α-PEA analog. The origin of this difference remains obscure (Scheme 44) [67].

### 6.9. Synthesis of Chiral Amino Alcohols and Diamines from α-PEA

**Scheme 45:**
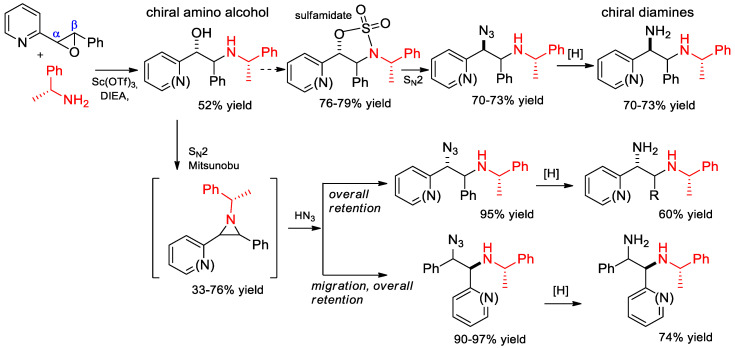
Synthesis of chiral β-amino alcohols and diamines.

Enantiomerically pure β-amino alcohols and diamines are important chiral building blocks and catalysts in asymmetric reactions. Recently, the synthesis of chiral 2-pyridinyl- and 6-(2,2′-bipyridinyl)-β-amino alcohols was reported [68]. The products were formed by ring opening of the corresponding 2-oxiranyl-compounds with chiral α-PEA and in the catalytic presence of scandium(III) trifluoromethanesulfonate (Scheme 45). The enantiomerically pure pyridine-β-amino alcohols were tested as chiral ligands in the zinc-catalyzed asymmetric aldol reaction with up to 55% *ee* outcome [68]. These compounds were further transformed into the respective diamines in a sequence of reactions involving the ring opening of the intermediate aziridines with HN_3_. Moreover, the enantiomerically pure β-amino alcohols were transformed into cyclic sulfamidates, and these compounds were reacted with sodium azide. After the Staudinger reduction, a series of diastereomeric *vic*-diamines with different stereo- and regiochemistry was obtained (Scheme 45) [69].

### 6.10. Removal of α-PEA Used as Chiral Auxiliary

Simple acidic extraction (aqueous 2M HCl or 10% H_2_SO_4_) allowed for recovery of chiral α-PEA after its use in optical resolution via diastereomeric salt formation, cf. Scheme 9. However, when α-PEA was employed as a chiral auxiliary, its liberation usually required splitting the corresponding chemical bonds. If an imide connection (a Schiff base) was applied, then again aqueous HCl was mostly enough for the α-PEA recovery. In some cases, the hydrolysis must be facilitated by the addition of NaHSO_3_ and thus the formed bisulfite allows for the removal of the carbonyl partner from the equilibrium, cf. Scheme 23. The cleavage of α-PEA-amides was usually carried out by hydrolysis in 6N HCl at 90 °C, cf. Scheme 29. For the splitting of the whole α-(methyl)benzyl moiety, catalyzed direct hydrogenolysis (Pd/C, H_2_, e.g., see, Scheme 30) or hydrogen-transfer debenzylation (see, Scheme 41) was used.

## 7. Synthesis and Application of Chiral Ligands with α-PEA Fragments in Asymmetric Reactions 

### 7.1. Enantioselective Arylation of N-Heteroaryl Aldimines and Hydrogenation Catalyzed by Ligands with PEA Fragments 

**Scheme 46:**
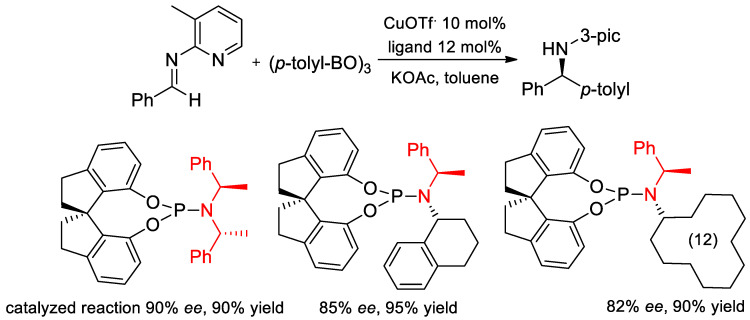
Screening chiral phosphoramidites for the arylation reaction.

An efficient stereoselective synthesis of chiral secondary amines attracts much interest because of the products’ potential pharmaceutical use. Recently, Zhou and coworkers described copper-catalyzed enantioselective arylation [70]. The catalytic reaction of *N*-azaaryl aldimines with arylboroxines was carried out in the presence of the prepared chiral phosphorus ligands with chiral α-PEA fragments. The best catalytic effects (up to 95% yield and 90% *ee*) were observed for the modular ligands with α-PEA moieties (Scheme 46).

**Scheme 47:**
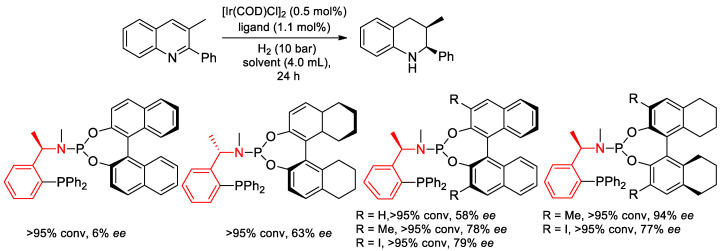
Unsymmetrical hybrid chiral phosphine–phosphoramidite ligands for asymmetric catalytic hydrogenation.

Other groups have synthesized a library of modular ligands with chiral α-PEA and phosphine-phosphoramidite fragments [71]. For the ligand synthesis, see below Scheme 50. The described dia- and enantioselective Ir-catalyzed hydrogenation of 3-alkyl-2-arylquinolines gave the product with a high yield and up to 94% *ee* (Scheme 47).

### 7.2. Catalyzed Enantioselective Addition of Organo-Zinc to Aldehydes

**Scheme 48:**
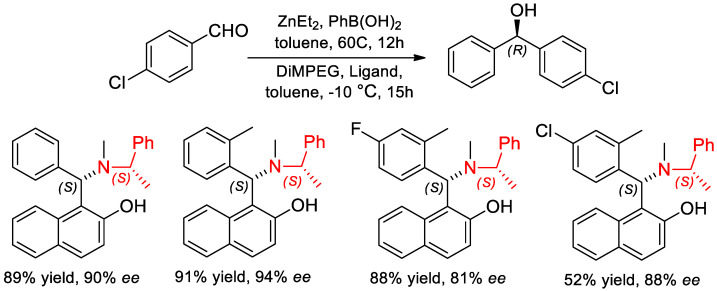
The asymmetric phenyl transfer reaction using various chiral aminonaphthol ligands.

The asymmetric addition of organo-zinc compounds to ketones or aldehydes leads to important chiral building blocks. Particularly, the catalytic transfer of the phenyl entity to aromatic aldehydes has attracted attention. For this purpose, new chiral tertiary aminonaphthol ligands were obtained in the reaction of 2-naphthol, (*S*)-α-PEA, and substituted benzaldehydes [72]. The ligands were screened in the Zn(II)-catalyzed asymmetric phenyl transfer from phenylboronic acid to the carbonyl of *p*-chlorobenzaldehyde. The reaction was run in toluene, in the presence of poly(ethylene glycol) dimethyl ether (DiMPG, 10 mol%). The arylation product was formed with high yield and enantioselectivity (up to 91%, 94% *ee*) (Scheme 48).

**Scheme 49:**
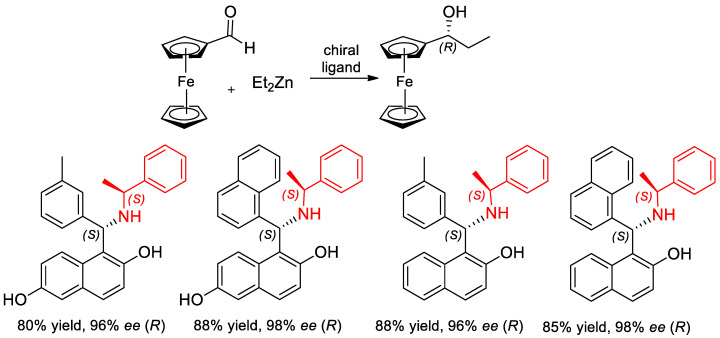
Addition of diethylzinc and alkynyl-Zn-reagents to aldehydes.

A series of chiral 1,3-aminonaphthols was synthesized using a Betti-type reaction between mono- or dihydroxy naphthalenes, aldehydes, and (*S*)-α-PEA [73]. Thus, the obtained modular ligands were applied in the enantioselective addition of Et_2_Zn and alkynyl-Zn to ferrocencarbaldehyde (Scheme 49). All the ligands with (*S*)-α-PEA fragments gave the *R* isomer of the product with good yield and enantioselectivity [73]. 

**Scheme 50:**
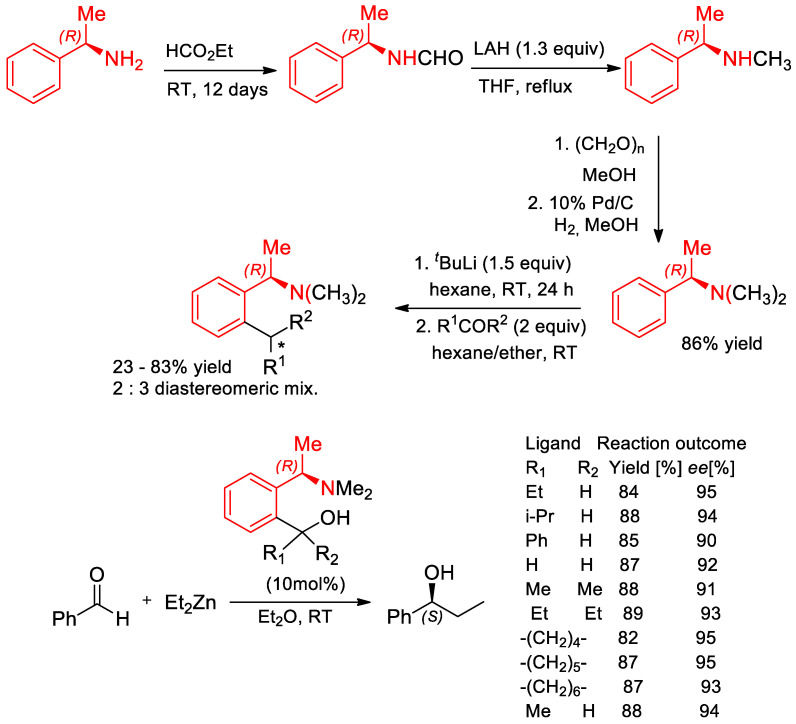
Enantioselective addition of diethylzinc to benzaldehyde.

Asami and coworkers synthesized a library of thirteen 1,4-amino alcohols, which were checked in the enantioselective addition of diethylzinc to benzaldehydes [74]. In all cases, 1-phenyl-1-propanol (*S* configuration) was obtained in high yield and enantioselectivity, regardless of the configuration at the second center of the ligand (Scheme 50). 

### 7.3. Asymmetric Hydrogenation Reactions 

**Scheme 51:**
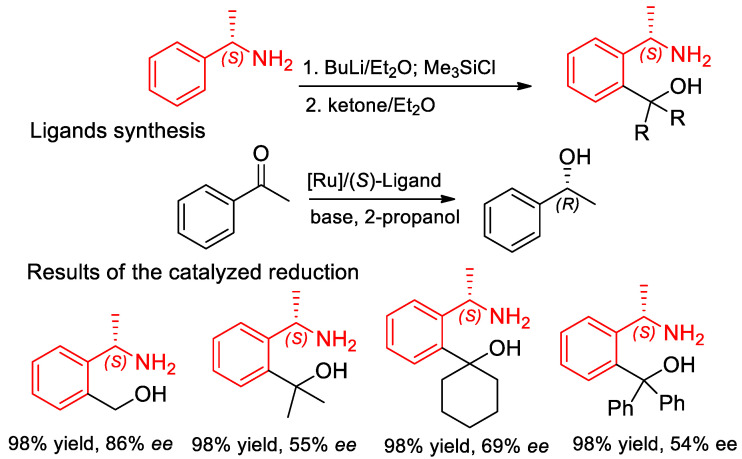
Ru-catalyzed asymmetric transfer hydrogenation of acetophenone.

Similar 1,4-amino alcohol ligands derived from (*S*)-α-PEA were prepared by a one-pot ortho-lithiation followed by a reaction with the carbonyls. Xiang-Ping Hu and coworkers used chiral amino alcohols as effective catalysts in the Ru-catalyzed asymmetric transfer hydrogenation of aromatic alkyl ketones [75]. The authors concluded that the amino alcohols’ structure influenced the conversion and enantioselectivity, and the less sterically demanding ligand gave higher enantioselectivity (Scheme 51).

**Scheme 52:**
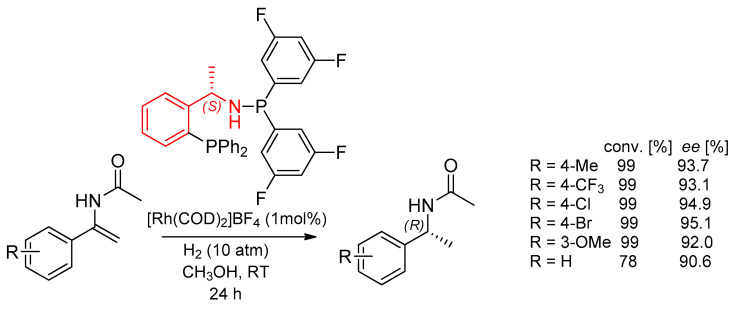
Rh-catalyzed asymmetric hydrogenation of olefins.

Catalytic hydrogenation is one of the most powerful and practical ways to access chiral compounds. Bidentate phosphorus ligands were used as efficient catalysts in this type of reaction. The Xiao-Mao Zhou group transformed enantiomeric α-PEA to prepare a series of chiral phosphine-aminophosphine ligands (Scheme 52) [76]. They used these ligands in the Rh-catalyzed asymmetric hydrogenation of olefins. Many different ligands were tested, and various functionalized olefins were transformed into the products with almost complete conversion and high enantioselectivity (Scheme 52).

**Scheme 53:**
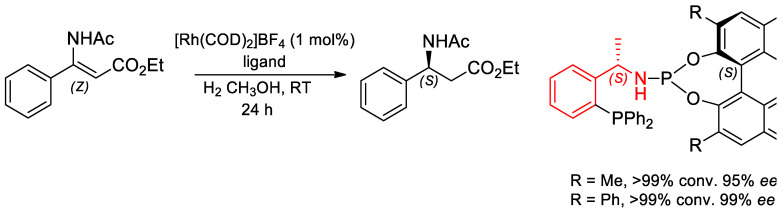
Chiral *P*,*N*-donating ligands tested in Rh-catalyzed asymmetric hydrogenation.

The same group reported another class of phosphine-phosphoramide chiral ligands with a 3,3′-substituted chiral binaphthyl fragment. These compounds were successfully tested in the catalytic reaction of asymmetric hydrogenation. Thus, ethyl (*Z*)-β-phenyl-β-(acetylamino)acrylate in methanol was reacted with gaseous hydrogen in the catalytic presence of Rh complexed with the new ligands. (*S*)-β-phenyl-β-(acetylamino)propanoate was obtained with excellent substrate conversion and enantioselectivity (Scheme 53) [77].

### 7.4. Deprotonations of Bicyclic Amino Ketones

**Scheme 54:**
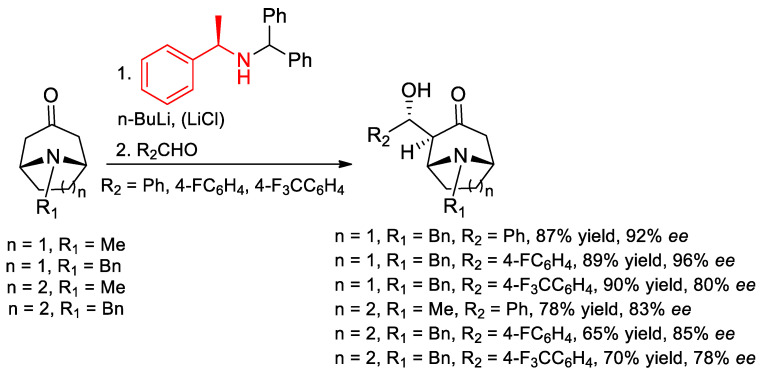
Deprotonations and aldol reactions of bicyclic amino ketones.

Asymmetric deprotonation combined with the aldol reaction allows for the enantioselective formation of a new C-C bond. Nodzewska and coworkers described a simple procedure for the synthesis of *N*-benzhydryl-derivatives of α-PEA via direct *N*-alkylation [78]. The lithium amides of this chiral secondary amine were used in the asymmetric deprotonation of tropinone analogs, followed by the aldol reaction with aldehydes giving products in high diastereomeric purities and enantiomeric excesses of 77 to 96% (Scheme 54) [78].

### 7.5. Polymerization of Rac-Lactide

**Scheme 55:**
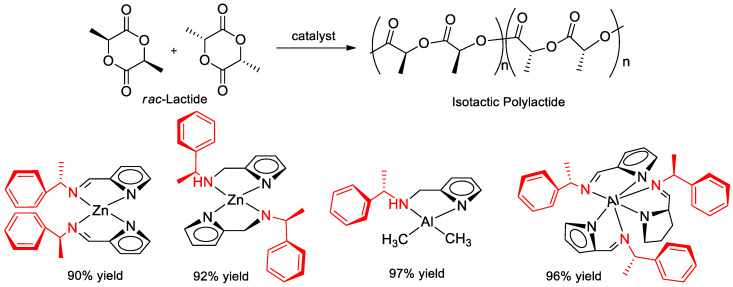
Synthesis of polylactide catalyzed by Zn and Al complexes.

Chiral ligands with α-PEA fragments have been synthesized and used to synthesize four novel chiral zinc and aluminum complexes. Their structures were confirmed by X-ray diffraction analyses. All complexes were active catalysts for the stereoselective polymerization of *rac*-lactide to isotactic polylactide, and the product was obtained in up to 97% yield (Scheme 55) [79].

## 8. α-PEA and Its Derivatives in Organocatalysis

### 8.1. Direct Use of α-PEA as an Organocatalyst

In some cases, small chiral molecules, as enantiomeric α-PEA itself, exhibited interesting catalytic properties. In the last decade, three organocatalytic reactions of this type have been reported. Specifically, the intramolecular tandem Michael–Henry reaction [80], α-fluorination of ketones [81], and Wittig rearrangement of ketones [82] were effectively carried out with α-PEA as an organocatalyst. 

**Scheme 56:**
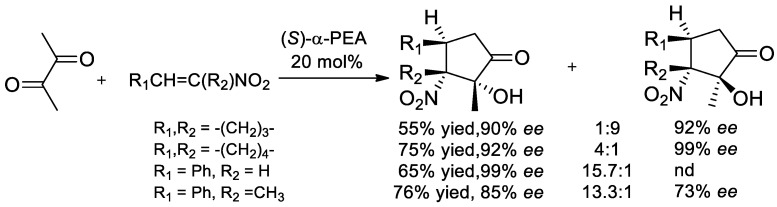
Reactions between 2,3-butanedione and conjugated nitroolefins catalyzed by α-PEA.

The first organocatalyzed reaction was the enantioselective formal [3  +  2] cyclization between 2,3-butanedione and conjugated nitroolefins in the presence of α-PEA (20 mol%). The reaction led to good yield and substantial diastereoselectivity of enantiomerically enriched 2-hydroxy-3-nitrocyclopentanones (Scheme 56) [80].

**Scheme 57:**
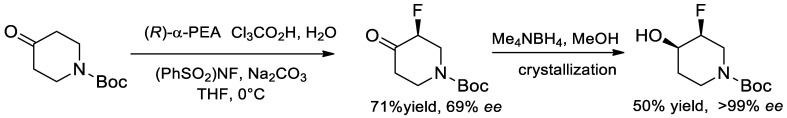
The enantioselective α-fluorination of 4-piperidones.

The second example refers to the enantioselective synthesis of both enantiomers of *cis*-1-Boc-3-fluoropiperidin-4-ol by fluorination catalyzed through chiral primary amines. The reaction catalyzed by commercially available amines, including α-PEA, resulted in similar enantioselectivity levels as attained with *epi*-9-aminoquinine. The piperidinols readily crystallized to the enantiopure material (Scheme 57). The products are important building blocks for the synthesis of pharmacologically active compounds [81]. 

**Scheme 58:**
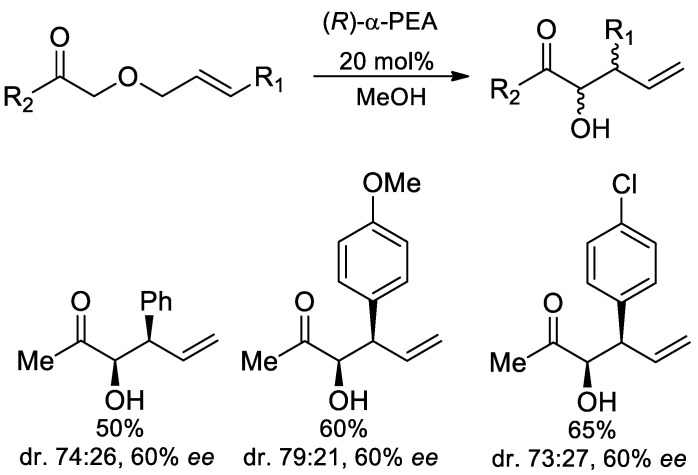
The products of the Wittig rearrangement catalyzed by α-PEA.

The third case involves the organocatalytic effect of α-PEA in sigmatropic [2,3]-Wittig rearrangement of allyloxyketones described in 2019 by the Šebesta group. Catalytic studies were carried out on many substrates, and numerous products were obtained in good yields and high enantiomeric excesses (Scheme 58) [82].

### 8.2. Enantioselective Aldol Addition

The products of direct asymmetric aldol reactions are molecular fragments of important molecules, often required by the pharmaceutical industry. This application creates persisting interest in the development of appropriate organocatalysts, thus avoiding metal catalysts, which are difficult to remove. 

**Scheme 59:**
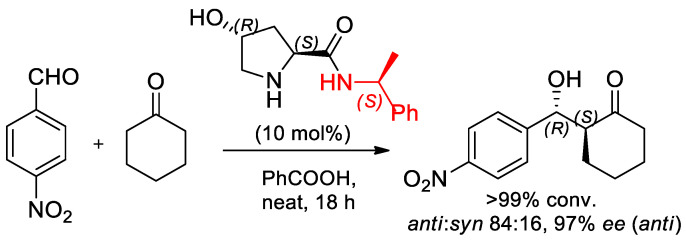
Asymmetric direct aldol reaction catalyzed by 4-hydroxy-(*S*)-prolinamide.

Thus, Yadav and Singh described the synthesis of trans-4-hydroxy-(*S*)-prolinamide with α-PEA fragments [83]. These organocatalysts were used with good results in the model aldol reaction between cyclohexanone and *p*-nirobenzaldehyde. The corresponding product was formed with almost complete conversion and high enantioselectivity up to 97% *ee* for the *anti* diastereoisomer (Scheme 59).

**Scheme 60:**
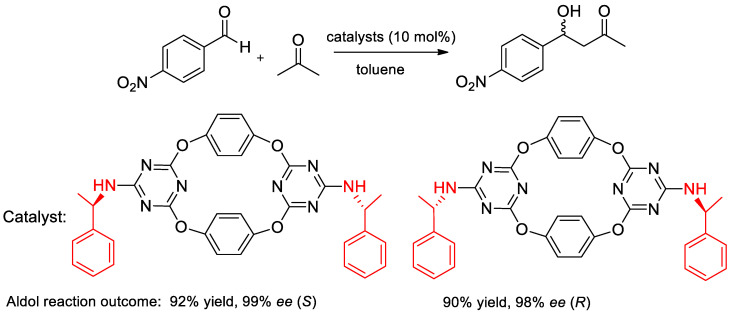
Tetraoxacalix[2]arene[2]triazine-based organocatalysts for enantioselective aldol reactions.

The next class of compounds devised as catalysts for the aldol reaction was chiral tetraoxacalix[2]arene[2]triazines. These organocatalysts based on chiral α-PEA were tested in the asymmetric aldol reaction of ketones with various aldehydes [84]. The adducts were obtained in 71–92% yields with good to excellent enantioselectivities (78–99%) (Scheme 60).

### 8.3. Enantioselective Micheal Addition

**Scheme 61:**
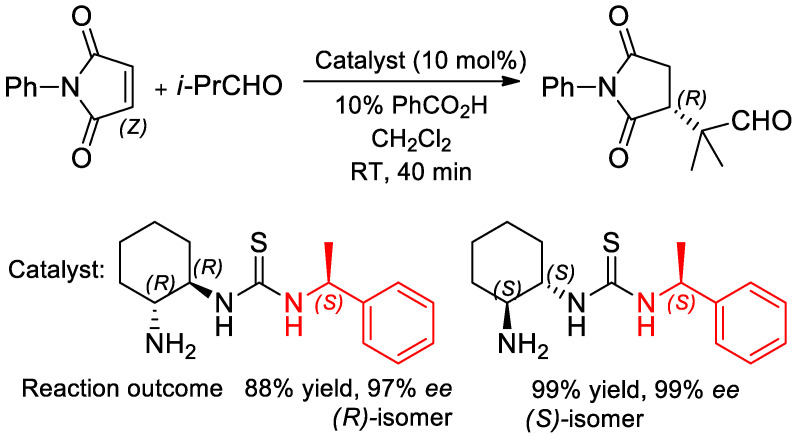
Catalytic evaluation in the Michael addition of isobutyraldehyde to *N*-phenylmaleimide with α-PEA-containing organocatalysts.

An interesting example of the enantioselective Michael reaction was recently described for chiral α-PEA-containing organocatalysts. The work published concerned the catalytic performance of chiral bifunctional thioureas *N*-substituted with methyl or trifluoromethyl groups in the Baylis–Hillman and Michael reactions [85]. In the presence of organocatalysts containing α-PEA fragments, the addition of isobutyraldehyde to *N*-phenylmaleimide gave the respective product with high yield and *ee* (Scheme 61). Noticeably, the observed asymmetric induction was governed by the configuration of the 1,2-diaminocyclohexane part.

## 9. Summary and Outlook

Despite the commercial availability of enantiomeric α-PEA, new advancements in its preparation and resolution were recorded. The lipase-catalyzed dynamic kinetic resolution was efficient with α-alkoxyacetic acid esters, and new synthetic techniques were used for these processes. Further, the effective resolution of other acidic compounds was carried out with enantiomeric α-PEA. For the amides of α-PEA, a self-disproportionation of enantiomers was documented. The use of α-PEA as a chiral auxiliary was mostly directed to the synthesis of medicinal substances and natural products, including 5-, 6-, and 7-membered chiral nitrogen heterocycles. It is noteworthy that even if the observed reaction diastereoselectivity was poor or medium, the α-PEA stereodifferentiating properties allowed simple separation of diastereomers by crystallization or chromatography. Particularly promising were numerous catalytic applications of modular chiral ligands and organocatalysts with α-PEA moieties.

Thus, the last decade witnessed successful syntheses using α-PEA-based chiral auxiliaries and encouraging outcomes of catalytic experiments. These recently achieved results stimulate further research, and rapid development in this field is anticipated.
